# Does where you live matter to your health? Investigating factors that influence the self-rated health of urban and rural Chinese residents: evidence drawn from Chinese General Social Survey data

**DOI:** 10.1186/s12955-017-0658-0

**Published:** 2017-04-21

**Authors:** Hongsheng Chen, Ye Liu, Zhenjun Zhu, Zhigang Li

**Affiliations:** 1 School of Architecture, Southeast University, Nanjing, 210096 China; 2 School of Geography and Planning, Sun Yat-sen University, Guangzhou, 510275 China; 3 School of Transportation, Southeast University, Nanjing, 210096 China; 4 School of Urban Design, Wuhan University, Wuhan, 430072 China

**Keywords:** Health disparity, Environmental quality, Green space, Physical exercise, Self-rated health, China

## Abstract

**Background:**

China’s rapid urbanization over the past decades has exacerbated the problems of environmental degradation and health disparities. However, few studies have analysed the differences between urban and rural residents in relation to how environmental quality impacts health outcomes. This study examines the associations between Chinese people's perceptions of environmental quality and their self-rated health, particularly focusing on differences between rural and urban residents in environment-health relationships.

**Methods:**

Using a logistic regression model and data from the 2013 Chinese General Social Survey (CGSS), a representative sample of data for 3,402 urban residents (46 ± 16 years) and 2,439 rural residents (48 ± 15 years) was analysed. The dependent variable used for the logistic regressions was whether or not respondents reported being healthy. Independent variables included respondents’ evaluations of the living environment, and how frequently they participated in physical activities. Interaction terms were employed to measure the moderating effects of physical exercise on the relationship between perceived environmental quality and health.

**Results:**

The percentage of healthy urban residents was significantly larger than that of healthy rural respondents (70.87% versus 62.87%). Urban respondents living in areas with sufficient green space were more likely to report good health (OR = 0.749, CI = [0.628, 0.895]), while rural respondents without reliable access to fresh water were more likely to report poor health (OR = 0.762, CI = [0.612, 0.949]). Urban respondents who were exposed to green spaces and exercised frequently were 21.6 per cent more likely to report good health than those who exercised infrequently (OR = 1.216, CI = [1.047, 1.413]). Those who lived in areas with insufficient green space and exercised frequently were 19.1 per cent less likely to report good health than those who exercised infrequently (OR = 0.805, CI = [0.469, 1.381]). No evidence suggested that physical exercise exerted a moderating effect on the relationship between exposure to air pollution and health.

**Conclusions:**

On average, urban residents have better health than rural residents. Among four indicators for low environmental quality (air pollution, lack of green spaces, water pollution, uncertain access to freshwater resources), green space was an important determinant of urban residents’ health status, while unreliable access to fresh water harmed rural residents’ health. Physical exercise moderated the effects of exposure to green spaces on urban residents’ health.

## Background

China has experienced rapid urbanization since the late 1970s, when economic reforms and opening up were introduced. Urbanization has had a substantial and complex influence on the health of Chinese people [1–4]. On the one hand, urbanization offers opportunities for health improvements, since people living in urban areas generally enjoy better living standards and health care than their rural counterparts. On the other hand, urbanization brings about many new health risk factors for Chinese people such as air pollution, sedentary lifestyles, and life stresses. Health disparities between rural and urban populations have grown substantially with the process of rapid urbanization over past decades [2, 5, 6], and the effects living environments have on the health outcomes of urban residents and rural residents differ significantly. China’s widening urban-rural health disparities motivate us to investigate this issue using nationwide representative survey data.

The relationships between the environment and human health differ substantially between China’s rural and urban areas. First, areas with a high level of urbanization often suffer from serious environmental pollution due to traffic emissions, high levels of energy consumption, and industrial structures [7–9]. Evidence has shown that air pollution has a negative impact on Chinese people’s health, and especially on urban residents’ health [10, 11]. Second, most people living in China’s high-density urban areas suffer from poor access to green spaces, while the problem of insufficient green spaces is less serious in low-density urban and rural areas [12, 13]. Third, the contamination of drinking water remains an important risk factor for rural residents’ health, since tap water is not common in some rural parts of China, and the water supply infrastructure in some rural areas is inadequate and dilapidated [14, 15]. For example, many “cancer villages” have appeared in rural China in recent years, as the result of severe water pollution [16]. In contrast, urban residents have better access to clean water, as China’s government gives priority to drinking water supplies in urban areas. Fourth, physical exercise may exert a moderating effect on the relationship between living environments and human health in urban areas [2]. For example, urban residents who frequently engage in outdoor exercise are more affected by their surrounding green spaces than those who have a sedentary lifestyle. In contrast, most rural residents engage in agricultural activities and do manual work every day, and their health is less affected by the frequency with which they participate in physical exercise.

However, most previous studies on the link between exposure to environmental pollutants and health outcomes in China have focused only on urban residents' health outcomes [17, 18]. Little attention has been given to differences between the effects of environmental quality on the health of urban and rural residents. The purpose of this study is to examine the factors that influence urban and rural residents’ self-rated health in China, with a particular focus on the effects of perceived environmental quality and physical activities on health. We use subjective evaluations of exposure to air pollution, lack of access to green spaces, water pollution, and shortages of fresh water as measures of environmental quality in the analysis of environment-health relationships.

Most previous studies have used area-level environmental quality indicators such as air quality indexes to study the association between environmental quality and health [17, 19]. Very few efforts have been made to unravel the link between individuals’ perceptions of their living environments and their self-rated health in Chinese urban and rural areas. The advantage of using individuals’ perceptions of their living environment—instead of area-level environmental quality indicators—for the study of environment-health relationships is that subjective indicators of environmental quality may be more telling indicators of individuals’ self-rated health than objective indicators [20, 21]. A handful of studies carried out in Chinese cities have demonstrated that residents’ perceptions of their neighborhoods’ physical and social environments were strongly associated with their self-rated physical health and mental wellbeing [22–24]. Therefore, it is worthwhile to examine the relationships between individuals’ perceptions of their living environments and their self-rated health in Chinese urban and rural areas.

China’s rapid urbanization has led to a transition from a physically demanding lifestyle to a sedentary lifestyle, thereby posing a great threat to Chinese people’s health [25, 26]. Regular moderate physical exercise has significant benefits for health: for one thing, physical exercise reduces the risks of infectious diseases such as the common cold, and chronic illnesses such as cardiovascular diseases and diabetes; for another, physical exercise improves mental wellbeing by relieving stress and regulating one’s mood [27]. Although a large body of literature has demonstrated a positive link between physical exercise and health, little research has evaluated this finding in relation to urban and rural residents. Moreover, studies in Western countries have found that physical exercise enhances people’s health indirectly, by increasing their opportunities for exposure to green spaces [28, 29]. It would be interesting to investigate this issue in China, and probe how urban and rural populations experience the moderating effects of physical exercise.

## Methods

### Data

This study analysed data from the 2013 Chinese General Social Survey (CGSS). The CGSS is China’s first nationwide, comprehensive, large-scale social survey project [30]. The 2013 CGSS data were collected from five types of regions (central urban areas, the edges of urban areas, urban-rural fringe areas, towns, and rural areas) using a multistage stratified probability proportional to size sampling technique. To focus the research and avoid ambiguities between urban and rural boundaries, we analysed data that were collected only in urban areas (central urban areas and the edges of urban areas) and only in rural areas. According to the actual proportions of urban and rural residents in China, the total sampling ratio of urban to rural residents was 6: 4. Shanghai, Beijing, and Tianjin (with urbanization rates of 88.02%, 86.30%, and 78.28%, respectively) did not report rural respondents’ data in the survey, since no rural neighbourhoods had been sampled. After missing values were removed, a total of 6,571 valid sample members were used for our analysis.

**Fig. 1:**
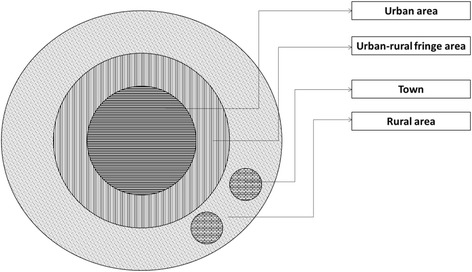
Schematic diagram of urban-rural spatial structure

Figure 1 depicts the ideal locations of urban areas, rural areas, and urban-rural fringe areas. There are differences in the living environments, economic structures, population composition, and lifestyles of China’s urban and rural areas, where urban land is mostly owned by the state [31]. Urban areas are characterized by high population densities, high building densities, low proportions of green spaces, and heavy traffic. Compared with rural areas, urban areas have higher levels of economic development and better infrastructure. Urban residents are mainly engaged in non-agricultural work. The ecological environments in rural areas are relatively good, and their population and building densities are low.

### Logistic regression model and variables

Using logistic regression analysis, this study estimated associations between residents’ perceptions of environmental quality and their self-rated health. The dependent variable was a binary variable for which a healthy group was coded as 1, and an unhealthy group was coded as 0. In the CGSS 2013, respondents were asked to evaluate their physical health using a five-point Likert scale ranging from 1–5 (1 = ‘very unhealthy’, 2 = ‘unhealthy’, 3 = ‘neither healthy nor unhealthy’, 4 = ‘healthy’, 5 = ‘very healthy’). We regrouped these five categories into two categories, namely an unhealthy group (1–3), and a healthy group (4–5).

Independent variables consisted of respondents’ evaluations of their living environments and how frequently they engaged in physical activities. For four objective indicators of environmental quality, respondents were asked to report how serious air pollution, water pollution, green space shortages, and freshwater resource shortages were in their place of residence, using a six-point Likert scale (1 = ‘very serious’, 2 = ‘moderately serious’, 3 = ‘somewhat serious’, 4 = ‘not too serious’, 5 = ‘not at all serious’, 6 = ‘no such problem’). We regrouped these six response categories into two—serious pollution/shortages (1–2), and no serious pollution/shortages (3–6)—based on the literal meanings of the response categories. The variable for physical exercise measured the frequency with which respondents engaged in physical exercise. In the survey questionnaire, respondents were asked to report the frequency with which they participated in physical exercise, using a five-point Likert scale (1 = ‘every day’, 2 = ‘several times a week’, 3 = ‘several times a month’, 4 = ‘several times a years’, 5 = ‘never’). We regrouped these five categories into two categories, namely, exercising frequently (1–3), and exercising infrequently (4–5), according to the distribution of sample members across five categories.

The control variables included age, gender, marital status, educational attainments, the logarithm of annual household income, hukou status, and participation in insurance schemes. Insurance schemes included basic medical insurance (BMI) schemes and personal commercial medical insurance (PCMI) schemes. State-administered BMI schemes are targeted at universal coverage and have three components: Urban Employee Basic Medical Insurance Scheme, Urban Resident Basic Medical Insurance Scheme, and the New Rural Cooperative Medical System. PCMI schemes are administered by business in the quasi-private and private sectors, and are targeted at those who are able to afford PCMI payments. We conducted a Variance Inflation Factor (VIF) test, and found no evidence of multicollinearity among the independent variables. We regressed self-rated health on living environments and physical activities separately for three groups of sample members as follows: all sample members, those living in urban areas, and those living in rural areas.

## Results

### Descriptive statistics

Table 1 provides descriptive statistics for the dependent and independent variables. Overall, the healthy group percentage of urban respondents (70.87%) was significantly larger than that among rural respondents (62.87%). However, as expected, the living environments in urban areas were significantly worse than in rural areas. The proportions of urban residents reporting serious air pollution, serious green space shortages, serious water pollution, and serious freshwater resource shortages were 50.56%, 31.53%, 43.50%, and 31.36%, respectively. In terms of physical exercise, the proportion of urban residents who engaged in frequent physical exercise was much larger than that of their rural counterparts (43.57% versus 14.40%). Regarding other individual characteristics, there were significant differences between rural and urban residents in their average ages, marital status, educational attainments, average annual household incomes, hukou status, and numbers with insurance coverage.

**Table 1:** Descriptive statistics of variables for rural and urban residents

	Rural residents		Urban residents		Test statistics/ Chi-square	*P *value
	Mean/Proportion	S.D.	Mean/Proportion	S.D.		
Dependent variable						
Self-rated physical health (%)					45.134^b^	0.000
Unhealthy	37.13		29.13			
Healthy	62.87		70.87			
Independent variables						
Evaluation of air pollution (%)					729.847^b^	0.000
Not serious	82.90		49.44			
Serious	17.10		50.56			
Evaluation of green space shortage (%)					448.839^b^	0.000
Not serious	91.23		68.47			
Serious	8.77		31.53			
Evaluation of water pollution (%)					255.300^b^	0.000
Not serious	76.09		56.50			
Serious	23.91		43.50			
Evaluation of freshwater resource shortage (%)					91.526^b^	0.000
Not serious	79.19		68.28			
Serious	20.81		31.65			
Doing physical exercise (%)					594.107 ^b^	0.000
Infrequently	85.60		56.43			
Frequently	14.40		43.57			
Control variables						
Age	48.04	15.23	46.41	15.85	4.098^a^	0.000
Gender (%)					0.028^b^	0.867
Male	53.61		53.40			
Female	46.39		46.60			
Marital status (%)					40.360^b^	0.000
Single, divorced, widowed, etc.	15.26		21.65			
Married	84.74		78.35			
Educational attainments (%)					1100^b^	0.000
Junior high school and below	85.11		43.54			
Senior high school	11.38		27.26			
Bachelor degree and above	3.51		29.20			
Annual household income (Yuan)	36413.10	45242.74	76545.49	90745.89	-20.435^a^	0.000
Hukou status (%)					213.380^b^	0.000
Non-local hukou	3.26		14.64			
Local hukou	96.74		85.36			
Insurance schemes (%)					174.131^b^	0.000
Have not joined any BMI and PCMI schemes	5.22		9.83			
Have joined both BMI and PCMI schemes	5.96		13.64			
Have joined BMI schemes but not PCMI schemes	88.21		74.78			
Have joined PCMI schemes but not BMI schemes	0.61		1.75			

^a^ *t*-Test statistics. ^b^Chi-square statistics

Table 2 summarizes the results of cross-tabulations conducted of urban and rural residents’ self-rated health and evaluations of their living environments. For urban residents, surprisingly, the healthy group reported significantly lower evaluations of living environments (air pollution, water pollution, and freshwater resource shortages) than the unhealthy group. Healthy rural residents reported a significantly poorer quality of environment (air pollution and less access to green spaces) than unhealthy rural residents, while the proportions of unhealthy groups that reported freshwater resource shortages were greater than those in the healthy groups. We suspect that these counterintuitive statistical results may be due to the fact that rich and well-educated people—who are generally healthier—are more concerned about the quality of their living environments. Further, in China, many rich and well-educated people are more likely to live in urban centre districts with low-quality living environments (e.g. traffic pollution, fewer green spaces), in order to be close to workplaces and have better access to public services. Therefore, a multivariate regression analysis is needed to confirm the different environment-health relationships of urban and rural residents.

**Table 2:** Cross-tabulation of urban and rural residents’ self-rated health and evaluations of their living environments

Independent variables	Urban residents				Rural residents			
	Unhealthy	Healthy	Chi-square	*P* value	Unhealthy	Healthy	Chi-square	*P *value
	Proportion	Proportion			Proportion	Proportion		
Evaluation of air pollution (%)			2.870	0.090			3.805	0.051
Not serious	51.50	48.60			84.84	81.77		
Serious	48.50	51.40			15.16	18.23		
Evaluation of green space shortage (%)			0.030	0.862			4.174	0.041
Not serious	68.67	68.39			92.75	90.33		
Serious	31.33	31.61			7.25	9.67		
Evaluation of water pollution (%)			2.742	0.098			1.286	0.257
Not serious	58.50	55.68			77.36	75.34		
Serious	41.50	44.32			22.64	24.66		
Evaluation of freshwater resource shortage (%)			13.943	0.000			5.932	0.015
Not serious	72.50	66.54			76.59	80.73		
Serious	27.50	33.46			23.41	19.27		

### Logistic regression of self-rated health

We used logistic regression models to examine the associations between respondents’ probabilities of being healthy, their evaluations of their living environments, and the frequency with which all respondents engaged in physical exercise (Table 3), the frequency with which urban respondents engaged in physical exercise (Table 4), and the frequency with which rural respondents engaged in physical exercise (Table 5). In Tables 3 to 5, Models 1, 4, and 7 estimate the main effects of living environments and physical exercise on self-rated health, Models 2, 5, and 8 estimate not only the main effects, but also the moderating effects of physical exercise on the relationship between air pollution and health, and Models 3, 6, and 9 estimate not only the main effects but also the moderating effects of physical exercise on the relationship between green spaces and health.

**Table 3:** Logistic regression analysis of self-rated physical health (All respondents)

	Model 1		Model 2		Model 3	
Evaluation of living environment	OR	95% Confidence Intervals	OR	95% Confidence Intervals	OR	95% Confidence Intervals
Air pollution (ref: not serious)	1.003	[0.869, 1.157]	1.029	[0.869, 1.219]	0.993	[0.861, 1.147]
Green space shortage (ref: not serious)	0.867*	[0.750, 1.002]	0.866*	[0.749, 1.000]	1.016	[0.847, 1.218]
Water pollution (ref: not serious)	1.047	[0.911, 1.203]	1.045	[0.909, 1.201]	1.044	[0.908, 1.200]
Freshwater resource shortage (ref: not serious)	0.986	[0.862, 1.129]	0.986	[0.861, 1.128]	0.986	[0.862, 1.129]
Physical activities						
Doing physical exercise (ref: infrequently)	1.092	[0.959, 1.243]	1.125	[0.951, 1.331]	1.216**	[1.047, 1.413]
Controlled variables						
Age	0.959***	[0.955, 0.962]	0.959***	[0.955, 0.962]	0.959***	[0.955, 0.962]
Gender (ref: male)	0.776***	[0.694, 0.868]	0.776***	[0.694, 0.869]	0.778***	[0.695, 0.870]
Marital status (ref: single, divorce, widowed.)	0.966	[0.829, 1.126]	0.967	[0.830, 1.126]	0.972	[0.834, 1.132]
Educational attainments (ref: junior high school and below)						
Senior high school	1.266***	[1.087, 1.475]	1.264***	[1.086, 1.473]	1.263***	[1.084, 1.470]
Bachelor degree and above	1.089	[0.908, 1.307]	1.088	[0.907, 1.305]	1.093	[0.910, 1.311]
Logarithmic of annual household income	1.349***	[1.264, 1.440]	1.349***	[1.264, 1.440]	1.344***	[1.259, 1.435]
Hukou status (ref: non-local hukou)	0.904	[0.733, 1.114]	0.905	[0.734, 1.115]	0.908	[0.736, 1.119]
Insurance schemes (ref: have joined neither schemes)						
Have joined both BMI and PCMI schemes	1.106	[0.839, 1.459]	1.110	[0.841, 1.463]	1.125	[0.853, 1.484]
Have joined BMI schemes but not PCMI schemes	1.075	[0.868, 1.332]	1.077	[0.869, 1.334]	1.087	[0.877, 1.347]
Have joined PCMI schemes but not BMI schemes	2.271**	[1.145, 4.504]	2.280**	[1.149, 4.525]	2.333**	[1.177, 4.627]
Interaction in relation to evaluation of living environment						
Doing physical exercise frequently × Serious air pollution (ref: Doing physical exercise frequently × No serious air pollution)			0.932	[0.727, 1.196]		
Doing physical exercise frequently × Serious green space shortage (ref: Doing physical exercise frequently × No serious green space shortage)					0.665***	[0.503, 0.879]
N	6571		6571		6571	
Log likelihood	-3684.023		-3683.871		-3679.925	
Chi-squared	881.210***		881.515***		889.405***	

Exponentiated coefficients; 95% confidence intervals in brackets; * *p* < 0.10, ** *p* < 0.05, *** *p* < 0.01

**Table 4:** Logistic regression analysis of self-rated physical health (Urban respondents)

	Model 4		Model 5		Model 6	
Evaluation of living environment	OR	95% Confidence Intervals	OR	95% Confidence Intervals	OR	95% Confidence Intervals
Air pollution (ref: not serious)	1.001	[0.828, 1.210]	1.063	[0.832, 1.358]	0.991	[0.820, 1.199]
Green space shortage (ref: not serious)	0.749***	[0.628, 0.895]	0.747***	[0.626, 0.892]	0.915	[0.720, 1.162]
Water pollution (ref: not serious)	1.068	[0.883, 1.292]	1.065	[0.881, 1.289]	1.067	[0.882, 1.291]
Freshwater resource shortage (ref: not serious)	1.145	[0.946, 1.387]	1.141	[0.942, 1.383]	1.145	[0.945, 1.387]
Physical activities						
Doing physical exercise (ref: infrequently)	1.063	[0.901, 1.255]	1.135	[0.896, 1.437]	1.229**	[1.004, 1.504]
Controlled variables						
Age	0.955***	[0.949, 0.961]	0.955***	[0.949, 0.960]	0.955***	[0.949, 0.960]
Gender (ref: male)	0.762***	[0.649, 0.894]	0.761***	[0.649, 0.893]	0.765***	[0.652, 0.898]
Marital status (ref: single, divorce, widowed.)	1.116	[0.911, 1.368]	1.116	[0.911, 1.368]	1.121	[0.914, 1.374]
Educational attainments (ref: junior high school and below)	1.000	[1.000, 1.000]	1.000	[1.000, 1.000]	1.000	[1.000, 1.000]
Senior high school	1.152	[0.942, 1.408]	1.150	[0.941, 1.406]	1.157	[0.947, 1.415]
Bachelor degree and above	0.963	[0.770, 1.204]	0.960	[0.768, 1.201]	0.966	[0.772, 1.209]
Logarithmic of annual household income	1.251***	[1.120, 1.398]	1.251***	[1.120, 1.398]	1.246***	[1.115, 1.393]
Hukou status (ref: non-local hukou)	0.890	[0.696, 1.137]	0.892	[0.697, 1.140]	0.891	[0.697, 1.139]
Insurance schemes (ref: have joined neither schemes)	1.000	[1.000, 1.000]	1.000	[1.000, 1.000]	1.000	[1.000, 1.000]
Have joined both BMI and PCMI schemes	1.295	[0.903, 1.856]	1.302	[0.908, 1.868]	1.317	[0.918, 1.890]
Have joined BMI schemes but not PCMI schemes	1.060	[0.796, 1.410]	1.062	[0.798, 1.414]	1.068	[0.802, 1.423]
Have joined PCMI schemes but not BMI schemes	2.576**	[1.037, 6.396]	2.595**	[1.044, 6.448]	2.659**	[1.071, 6.604]
Interaction in relation to evaluation of living environment						
Doing physical exercise frequently × Serious air pollution (ref: Doing physical exercise frequently × No serious air pollution)			0.884	[0.641, 1.219]		
Doing physical exercise frequently × Serious green space shortage (ref: Doing physical exercise frequently × No serious green space shortage)					0.655**	[0.467, 0.918]
N	3402		3402		3402	
Log likelihood	-1828.113		-1827.829		-1825.087	
Chi-squared	443.354***		443.923***		449.406***	

Exponentiated coefficients; 95% confidence intervals in brackets; * *p* < 0.10, ** *p* < 0.05, *** *p* < 0.01

**Table 5:** Logistic regression analysis of self-rated physical health (Rural respondents)

	Model 7		Model 8		Model 9	
Evaluation of living environment	OR	95% Confidence Intervals	OR	95% Confidence Intervals	OR	95% Confidence Intervals
Air pollution (ref: not serious)	0.967	[0.736, 1.270]	1.025	[0.766, 1.372]	0.973	[0.740, 1.278]
Green space shortage (ref: not serious)	1.264	[0.900, 1.777]	1.260	[0.897, 1.770]	1.157	[0.801, 1.671]
Water pollution (ref: not serious)	1.020	[0.803, 1.295]	1.018	[0.801, 1.292]	1.018	[0.802, 1.293]
Freshwater resource shortage (ref: not serious)	0.762**	[0.612, 0.949]	0.762**	[0.613, 0.949]	0.761**	[0.611, 0.947]
Physical activities						
Doing physical exercise (ref: infrequently)	1.128	[0.859, 1.481]	1.218	[0.898, 1.653]	1.068	[0.803, 1.421]
Controlled variables						
Age	0.962***	[0.956, 0.968]	0.962***	[0.956, 0.968]	0.962***	[0.955, 0.968]
Gender (ref: male)	0.779***	[0.651, 0.932]	0.780***	[0.652, 0.934]	0.780***	[0.652, 0.934]
Marital status (ref: single, divorce, widowed.)	0.837	[0.640, 1.094]	0.838	[0.641, 1.096]	0.837	[0.640, 1.094]
Educational attainments (ref: junior high school and below)	1.000	[1.000, 1.000]	1.000	[1.000, 1.000]	1.000	[1.000, 1.000]
Senior high school	1.261	[0.917, 1.733]	1.260	[0.916, 1.732]	1.261	[0.917, 1.733]
Bachelor degree and above	1.519	[0.826, 2.793]	1.519	[0.826, 2.794]	1.506	[0.819, 2.772]
Logarithmic of annual household income	1.428***	[1.299, 1.570]	1.429***	[1.300, 1.570]	1.429***	[1.300, 1.570]
Hukou status (ref: non-local hukou)	1.142	[0.652, 2.000]	1.136	[0.648, 1.989]	1.151	[0.657, 2.017]
Insurance schemes (ref: have joined neither schemes)	1.000	[1.000, 1.000]	1.000	[1.000, 1.000]	1.000	[1.000, 1.000]
Have joined both BMI and PCMI schemes	0.805	[0.467, 1.388]	0.801	[0.465, 1.382]	0.805	[0.467, 1.389]
Have joined BMI schemes but not PCMI schemes	1.051	[0.697, 1.584]	1.051	[0.697, 1.585]	1.050	[0.696, 1.582]
Have joined PCMI schemes but not BMI schemes	1.074	[0.298, 3.879]	1.086	[0.301, 3.922]	1.079	[0.299, 3.893]
Interaction in relation to evaluation of living environment						
Doing physical exercise frequently × Serious air pollution (ref: Doing physical exercise frequently × No serious air pollution)			0.683	[0.353, 1.319]		
Doing physical exercise frequently × Serious green space shortage (ref: Doing physical exercise frequently × No serious green space shortage)					1.770	[0.686, 4.566]
N	2439		2439		2439	
Log likelihood	-1447.955		-1447.320		-1447.220	
Chi-squared	334.624***		335.893***		336.093***	

Exponentiated coefficients; 95% confidence intervals in brackets; * *p* < 0.10, ** *p* < 0.05, *** *p* < 0.01

Model 1 shows that respondents living in areas with insufficient green spaces were 13.3 per cent less likely to report good health (OR = 0.867, CI = [0.750, 1.002]). This finding indicates that exposure to green space is beneficial to health. However, no evidence showed that respondents’ evaluations of air pollution, water pollution, and freshwater resource shortages were linked to their self-rated health. Doing physical exercise frequently was not significantly associated with good health. As for control variables, the odds of reporting poor self-rated health increased with age (OR = 0.959, CI = [0.955, 0.962]), and decreased with increasing annual household incomes (OR = 1.349, CI = [1.264, 1.440]). Females were 22.4 per cent less likely than males to report being healthy (OR = 0.776, CI = [0.694, 0.868]). Respondents with senior high school education or above were 26.6 per cent more likely to report being healthy than those with a junior high school education or less (OR = 1.266, CI = [1.087, 1.475]). Respondents who had joined PCMI schemes but not BMI were 1.27 times more likely than others to report being in good health (OR = 2.271, CI = [1.145, 4.504]).

Model 2 illustrates that the coefficient of interaction term between the frequency of engaging in physical exercise and being exposed to air pollution was not significant. This indicated that physical exercise played no role in moderating the relationship between air pollution and self-rated health. Model 3 suggests that respondents who were exposed to green spaces and did physical exercise frequently were 21.6 per cent more likely than those who did exercise infrequently, to report good health (OR = 1.216, CI = [1.047, 1.413]), but respondents who did physical exercise frequently and lived in areas with insufficient green space were 19.1 per cent less likely than those who did exercise infrequently, to report good health (OR = 0.665 * 1.216 = 0.809, CI = [0.527, 1.242]).

Table 4 summarizes the results from regression analyses of data for respondents living in urban areas only. Model 4 shows that respondents living in areas with serious green space shortages were 25.1 per cent less likely than those living in areas with abundant green spaces to report good health (OR = 0.749, CI = [0.628, 0.895]). Other variables of living environments and the variable of engaging in physical activities were not significant. Among the control variables, similar to model 1, the odds of reporting poor self-rated health increased with age (OR = 0.955, CI = [0.949, 0.961]) and decreased with increasing annual household income (OR = 1.251, CI = [1.120, 1.398]). Females were less likely to report good health than males (OR = 0.762, CI = [0.649, 0.894]). Respondents who had joined PCMI schemes but not BMI were more likely to report good health than others (OR = 2.576, CI = [1.037, 6.396]). In Models 5 and 6, we examined whether the relationship between self-rated health and environmental quality was moderated by the frequency of engaging in physical exercise. The corresponding results were similar to those for Models 2 and 3: doing physical exercises frequently had no moderating effect on the relationship between exposure to air pollution and health, but did have a significant moderating effect on the relationship between green space exposure and health. Urban respondents who were exposed to green spaces and frequently engaged in physical exercise were 22.9 per cent more likely to report good health than those who exercised infrequently (OR = 1.229, CI = [1.004, 1.504]), but those who engaged in physical exercise frequently and lived in areas with insufficient green spaces were 19.5 per cent less likely than those who exercised infrequently to report good health (OR = 0.655 * 1.229 = 0.805, CI = [0.469, 1.381]).

Table 5 shows the results of the regressions for respondents living in rural areas. In model 7, respondents living in areas that lacked freshwater resources were 23.8 per cent less likely to report good health (OR = 0.762, CI = [0.612, 0.949]). Other variables of perceived environmental quality were not significant. This finding indicated that in rural areas freshwater resource shortages were a more influential factor than green space shortages, air pollution, and water pollution. For control variables, older respondents were more likely to report poor health (OR = 0.962, CI = [0.956, 0.968]). Respondents that were better off financially were more likely to report good health (OR = 1.428, CI = [1.299, 1.570]). Females were more likely to report poor health than males (OR = 0.779, CI = [0.651, 0.932]). Models 8 and 9 showed that interactions between air pollution and physical exercise, and interactions between green spaces and physical exercise were not statistically significant. There is no evidence suggesting that the relationship between rural residents’ perceptions of air pollution/green spaces and their health were moderated by the frequency with which they engaged in physical exercise.

## Discussion

Consistent with the existing literature on China’s urban-rural health disparities [5], we found that the health levels of urban residents were significantly higher than those of rural residents. Although numerous studies have attributed urban-rural health disparities to the unbalanced economic development that has occurred between urban and rural areas [5, 32], few studies have focused on the disparities of environment-health relationships between China’s urban and rural areas. Our findings suggest that where one lives (urban or rural areas) matters with regard to the determinants of one’s health. Using the 2013 CGSS data, we found that green spaces were an important determinant of urban residents’ health, and freshwater resource shortages were harmful to rural residents’ health. The differences between urban and rural areas in terms of the environmental determinants of their populations’ health reflect the fact that urbanization has dramatically changed Chinese people’s health-related behaviours and living environments [1, 3].

Exposure to green environments plays an important role in preventing chronic and non-communicable illnesses (e.g. cardiovascular diseases), through mechanisms that include stress reduction, attention restoration, and prolonged physical activities [33]. The shortage of green space in urban areas is primarily due to the unsustainable development of China’s cities. Most urban land in China is owned and controlled by the state, and land leasing has become the main source of revenue for local governments [34]. Therefore, local authorities are keen to promote urbanization and expand urban areas rapidly, but lack the motivation to allocate land for public green spaces. Moreover, in order to ensure the development of real estate, large tracts of wild green space in suburban areas (e.g. urban forest parks, grasslands) have been requisitioned for real estate development [35]. Additionally, the imbalance in urban and rural economic development has led to a surge in migrations from rural to urban areas [36]. Under these circumstances, the available urban green space per capita keeps declining, and the shortage of green space becomes a pressing problem for urban residents.

Freshwater resource shortages have had a significant negative impact on rural residents’ health [14, 37]. There are several reasons for this. First, it is related to the uneven development of China’s urban and rural areas. Water infrastructure is inadequate and underdeveloped in rural areas, since public investments in urban areas’ infrastructure were given priority [38]. Second, water-use efficiency was very low in rural areas [39]. This low efficiency was due to inefficient irrigation practices and water pollution (e.g. chemical fertilizer pollution). Third, water pollution became the main driver of freshwater resource shortages. Some scholars have found that surface water quality deteriorates in rural areas as a result ineffective environmental policies and management [40]. For example, the excessive use of chemical fertilizers and pesticides has led to the contamination of underground water [41]; the proliferation of small rural factories with little capacity for curbing water pollution is another example [42].

Our findings also suggest that physical activities moderated the relationship between urban residents’ self-rated health and their exposure to urban green spaces. Physical exercise promoted urban residents’ health when they lived in areas with abundant green spaces. However, physical exercise was not significantly related to rural residents’ self-rated health. This was probably because most rural residents did agricultural work for a living. Agricultural work in China requires intensive manual labour, as the degree of mechanization remains at a low level in the agricultural sector. Occupational physical activities may act as a substitute for physical exercise. Therefore, rural residents rarely participated in physical exercise, and their health was not significantly influenced by their participation in physical exercise.

Our study encountered some limitations. First, we used respondents’ subjective evaluations to measure the quality of the living environments, and therefore faced the risk of selective bias in the models’ results. For example, people who reported poor health were likely to report living in an area with serious environmental pollution, while healthier people were likely to report no exposure to environmental pollution. If this is the case, our models will have underestimated the effects of environmental exposure on self-rated health. Second, compared with urban residents, rural residents are more likely to overrate or underrate the environmental pollution problems at their places of residence, since they have insufficient means and less ability to acquire real-time pollution information (e.g. Air Quality Index). In recent years, however, there has been a rise in the rate at which the Internet and mobile phones are used in rural China. With the popularization of the Internet and mobile phones, it becomes easier for Chinese rural residents to acquire accurate health and environmental information [43, 44]. With the intensification of China’s rural environmental pollution, rural residents’ environmental awareness is awakening, and rural residents have a growing list of environmental requirements and have become increasingly sensitive to environmental pollution [45, 46]. Third, CGSS was carried out in the form of a cross-sectional survey rather than a longitudinal survey. We were therefore not able to control for unobserved individual characteristics that were consistent over time (e.g. genetic factors and family backgrounds). Failure to control these factors may have led to a bias in our estimates of the effects of environmental quality and physical exercise on health.

## Conclusions

Using logistic regression to analyse data from the Chinese General Social Survey (CGSS), this study examined the association between perceived environmental quality and in China’s urban and rural residents’ self-rated health. Model results showed that urban residents generally had better health than rural residents. When all other variables were controlled, urban residents’ health was negatively associated with their evaluation of green space shortages, while rural residents’ health was negatively related to freshwater resource shortages. Model results further suggested that physical activities moderated the association between urban residents’ exposure to green spaces and their health, and that doing physical exercises frequently enhanced the positive effects of green spaces. No evidence suggested that physical exercise exerted a moderating effect on the relationship between exposure to air pollution and health.

There is a remarkable health gap between China’s urban and rural residents. Such an urban-rural divide indicates an imbalance in development that favours urban areas. China’s rapid urbanization has led to serious environmental problems, and shaped Chinese people’s health-related behaviours. These emerging health risk factors pose an unparalleled challenge to health promotion in China. This study suggests that there is an urgent need to reduce health disparities between urban and rural areas. Importantly, it should be noted that the adverse effects of environmental hazards on health vary between urban and rural areas. Different health promotion measures are needed for different areas. For urban areas, governments are advised to implement strategies that will increase the supply of urban green spaces and encourage residents to adopt a healthy lifestyle. In rural areas, the highest priority should be given to increasing public investments in water infrastructures and improving the effectiveness of environmental regulations.
